# Turning the Stimulus On and Off Changes the Direction of α Traveling Waves

**DOI:** 10.1523/ENEURO.0218-20.2020

**Published:** 2020-11-18

**Authors:** Zhaoyang Pang (庞兆阳), Andrea Alamia, Rufin VanRullen

**Affiliations:** 1 Centre de Recherche Cerveau et Cognition (CerCo), Centre National de la Recherche Scientifique, Université de Toulouse, Toulouse 31052, France; 2 Artificial and Natural Intelligence Toulouse Institute (ANITI), Toulouse 31000, France

**Keywords:** α oscillations, predictive coding, traveling waves, visual processing, waves propagating direction

## Abstract

Traveling waves have been studied to characterize the complex spatiotemporal dynamics of the brain. Several studies have suggested that the propagation direction of α traveling waves can be task dependent. For example, a recent electroencephalography (EEG) study from our group found that forward waves (i.e., occipital to frontal, FW waves) were observed during visual processing, whereas backward waves (i.e., frontal to occipital, BW waves) mostly occurred in the absence of sensory input. These EEG recordings, however, were obtained from different experimental sessions and different groups of subjects. To further examine how the waves’ direction changes between task conditions, 13 human participants were tested on a target detection task while EEG signals were recorded simultaneously. We alternated visual stimulation (5-s display of visual luminance sequences) and resting state (5 s of black screen) within each single trial, allowing us to monitor the moment-to-moment progression of traveling waves. As expected, the direction of α waves was closely linked with task conditions. First, FW waves from occipital to frontal regions, absent during rest, emerged as a result of visual processing, while BW waves in the opposite direction dominated in the absence of visual inputs, and were reduced (but not eliminated) by external visual inputs. Second, during visual stimulation (but not rest), both waves coexisted on average, but were negatively correlated. In summary, we conclude that the functional role of α traveling waves is closely related with their propagating direction, with stimulus-evoked FW waves supporting visual processing and spontaneous BW waves involved more in top-down control.

## Visual Abstract

**Figure:**
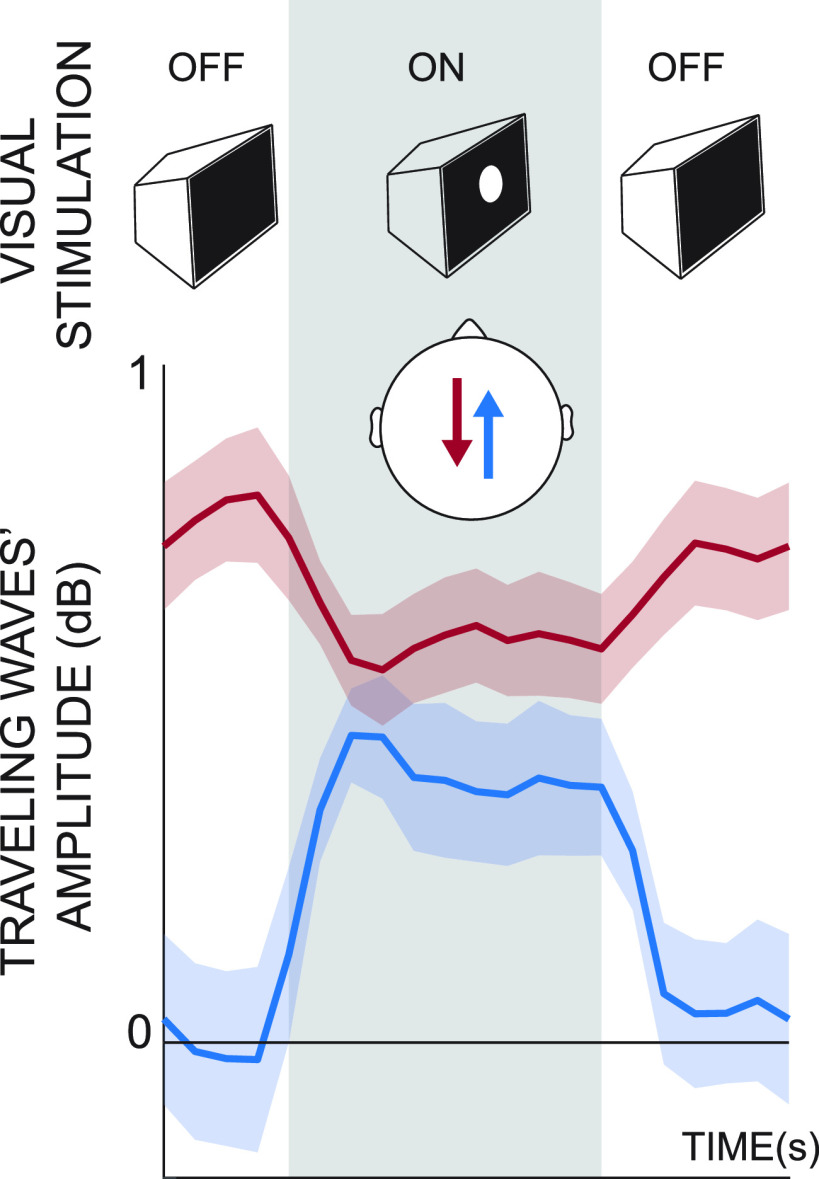

## Significance Statement

Several electroencephalography (EEG) studies have suggested that the propagation direction of α traveling waves can be task dependent; however, these recordings were obtained from different experimental sessions and different groups of subjects. Here, we conducted a human EEG experiment with both visual processing and resting state combined into each single trial. Forward waves (FW waves) from occipital to frontal regions, absent during rest, emerged as a result of visual processing, while backward waves (BW waves) dominated in the absence of visual inputs. Importantly, during visual processing, both FW and BW α waves were present and modulated by stimulation type (static or dynamic), but they were negatively correlated over time.

## Introduction

Neural oscillations at various temporal frequencies are ubiquitous in the human brain, and in the spatial domain, an increasing number of studies suggest that these oscillations could be organized as traveling waves across brain regions. The existence of traveling waves has been reported across multiple species (Ermentrout and Kleinfeld, 2001; Sato et al., 2012), at differing scales of measurements (Muller et al., 2018), and under various stimulation conditions (Nauhaus et al., 2012; Sato et al., 2012). Since the propagation of the traveling waves covers highly distributed brain regions, researchers have attempted to relate their functional significance to various aspects of the traveling waves. In particular, the directionality of traveling waves is believed to be functionally relevant (Klimesch et al., 2007a; Fellinger et al., 2012; Patten et al., 2012; Bahramisharif et al., 2013). For example, Halgren and colleagues showed that, during wakefulness with open or closed eyes, α oscillations recorded with intracortical electrodes from epilepsy patients propagated from antero-superior cortex toward postero-inferior occipital poles (Halgren et al., 2019). However, in another intracortical study (Zhang et al., 2018), when subjects were instructed to complete a visual memory task, traveling waves in the θ-α band (2–15 Hz) propagated from posterior to anterior brain areas. This apparent forward direction of traveling waves was also reported in studies of so-called “perceptual echoes,” which constitute a direct index of sensory processing (VanRullen and Macdonald, 2012). Participants were stimulated with random (white-noise) luminance sequences, and the resulting impulse response function showed a long-lasting 10-Hz oscillation (or perceptual echo); importantly, the spatial distribution of echo phase was organized as a traveling wave propagating from posterior to frontal sensors (Alamia and VanRullen, 2019; Lozano-Soldevilla and VanRullen, 2019). It thus seems that the directionality of traveling waves could be task dependent. To clarify the traveling direction with respect to various experimental conditions, a recent study (Alamia and VanRullen, 2019) from our group simulated α oscillations as a cortical traveling wave within a predictive coding framework. The predictive coding framework characterizes a hierarchical network where higher levels of brain regions predict the activity of lower levels, and the unexplained residuals (i.e., prediction errors) are passed back to higher layers. The study revealed that the recursive nature of predictive coding not only gave rise to α oscillations but also explained their propagating dynamics. Remarkably, when feeding with visual inputs (e.g., white noise), simulated α oscillations propagated from lower level to higher level, while simulating resting state gave rise to feedback waves.

The computational study suggests that the directionality of traveling waves could be closely linked with task conditions (visual processing vs rest state) and is supported by human electroencephalography (EEG) studies where participants were instructed to monitor a visual luminance sequence or keep their eyes closed. However, those human experiments were conducted separately within different experimental sessions and different groups of participants, and it is thus difficult to infer a direct relationship between the task condition and waves’ direction. To verify the predictions of the computational work and to systematically examine how the waves’ direction changes from one task condition to another, the current EEG study was designed to incorporate stimulus-on periods (visual processing) and stimulus-off periods (resting state) within each single trial, by which we could trace the moment-to-moment changes of the waves’ direction caused by task conditions in a consistent way.

## Materials and Methods

### Participants

A total of 14 subjects participated in this experiment. One subject was rejected because of a technical problem during the experimental recording, leaving 13 subjects (six females; mean age 25.57, range 21–31; two left-handed) for inclusion in the analysis. All participants reported no history of epileptic seizures or photosensitivity and they had normal or corrected to normal vision. Before starting the experiment, all participants gave written informed consent as specified by the Declaration of Helsinki. The study was performed under the guidelines for research according to author’s research institute at the Centre de Recherche Cerveau et Cognition and the protocol was approved by the committee Comité de protection des Personnes Sud Méditerranée 1 (ethics approval number N° 2016- A01937-44).

### Stimuli generation

**Figure 1.**
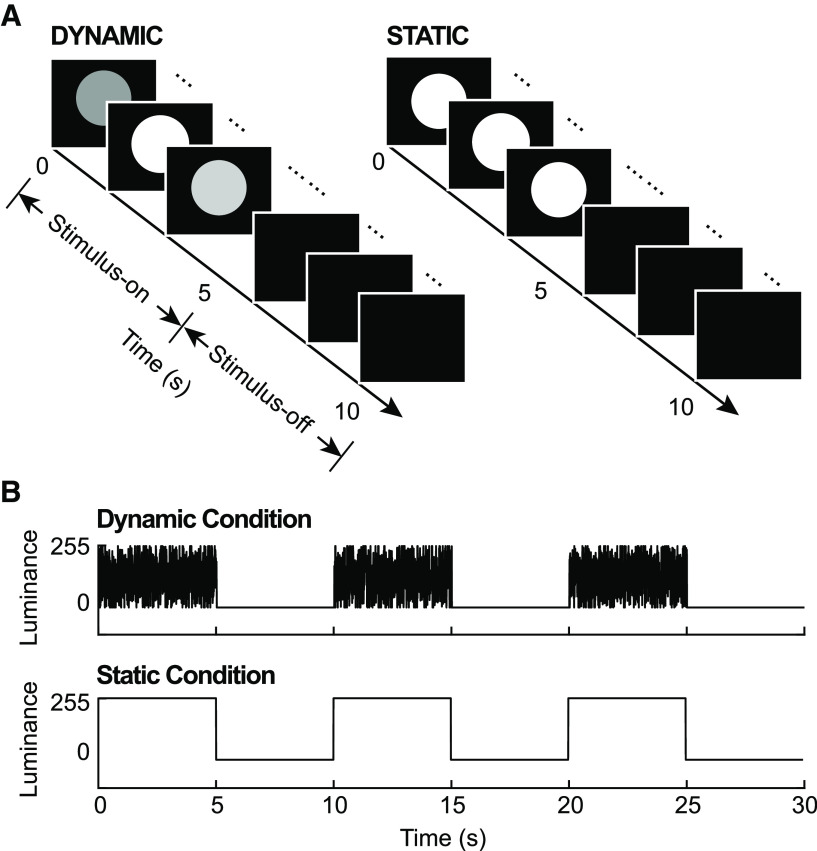
Experiment design. ***A***, Two types of trials were included in this experiment. For static trials, the luminance of visual input was held constant at a value of 255 (full contrast), while for dynamic ones, the luminance changed randomly from 0 to 255 on each screen refresh. In both cases, luminance sequences were displayed for the first 5 s (stimulus-on period), then followed by 5 s of blank screen (stimulus-off period). ***B***, Schematic diagrams of two subblocks for both dynamic and static conditions. Each subblock contained three identical trials, which made up a 30-s-long time course.

Visual stimuli were generated using MATLAB scripts and presented using the Psychophysics Toolbox (Brainard, 1997). The stimuli were displayed on a cathode ray monitor in a dark room, positioned 57 cm from the subjects, with a refresh rate of 160 Hz and a resolution of 800 × 600 pixels. We used two types of visual luminance sequences (Fig. 1) as visual inputs: dynamic (or white-noise) and static stimulation. For the white-noise sequences, the power spectrum was normalized to have equal power at all frequencies (up to 80 Hz). The resulting luminance of white-noise sequences ranged from black (0.1 cd/m^2^) to white (59 cd/m^2^), whereas the static ones were held constant with full contrast (59 cd/m^2^). Luminance sequences were displayed for 5 s within a disk of 3.5° radius which was centered at 7.5° above a center white dot on a black background.

### Experimental design

Subjects were instructed to perform a visual detection task. During the experiment, three identical trials (either static or dynamic) were displayed in a row, grouped into a subblock (Fig. 1*B*). Before each subblock, a green center dot was displayed until subjects pressed the space bar to indicate their readiness. The green dot then disappeared and was followed by those three trials after a time interval of 200–300 ms. A prototypical trial started (Fig. 1*A*) with 5 s of luminance sequences (either dynamic or static) in a disk above a white fixation dot at the center of the screen and then 5 s of blank screen. That is, each trial contained a stimulus-on period and a stimulus-off period, which allowed us to investigate the moment-to-moment changes of traveling waves when shifting from one task condition to another. Observers were asked to keep their fixation throughout the trial. Also, during visual stimulation (stimulus-on period), observers needed to covertly attend the disk to detect a brief square target (decreased luminance) inside the disk.

Two types of trials lead to two corresponding subblocks, dynamic or static, which were presented alternatively and also counterbalanced within subjects. Targets (1 s) appeared at a random time (uniform distribution) from 0.25 s after the onset to 0.25 s before the offset of luminance sequences on a random 20% of trials. The square target luminance was adjusted according to each subject by a staircase procedure using the Quest function (Watson and Pelli, 1983) to ensure 80% detection rate. In dynamic trials, the luminance of the square target fluctuated according to the white-noise sequences, but with a lower contrast compared with the rest of the disk. The experiment was composed of five sessions of 10 experimental blocks of six trials (i.e., two subblocks) each, with a total duration of ∼1 h.

### EEG recording and preprocessing

Continuous brain activity was recorded from the subjects using a 64-channel active BioSemi EEG system, with 1024-Hz digitizing sample rate and three additional ocular electrodes. Custom scripts in the EEGlab toolbox (Delorme and Makeig, 2004) were applied to the pre-processing steps, during which both target-present and target-absent trials were included. We first rejected the noisy channels and then the data were offline down-sampled to 160 Hz. In order to remove power line artefacts, a notch filter (47–53 Hz) was applied. We applied an average-referencing and removed slow drifts by applying a high-pass filter (>1 Hz). Data epochs were created around −0.5 to 10 s around the trial onset, and EEG activity was corrected by subtracting the baseline activity from −0.5 to 0 s before trial onset. Finally, the data were screened manually for eye movements, blinks and muscular artefacts and whole epochs were rejected as needed.

### Wave quantification

**Figure 2.**
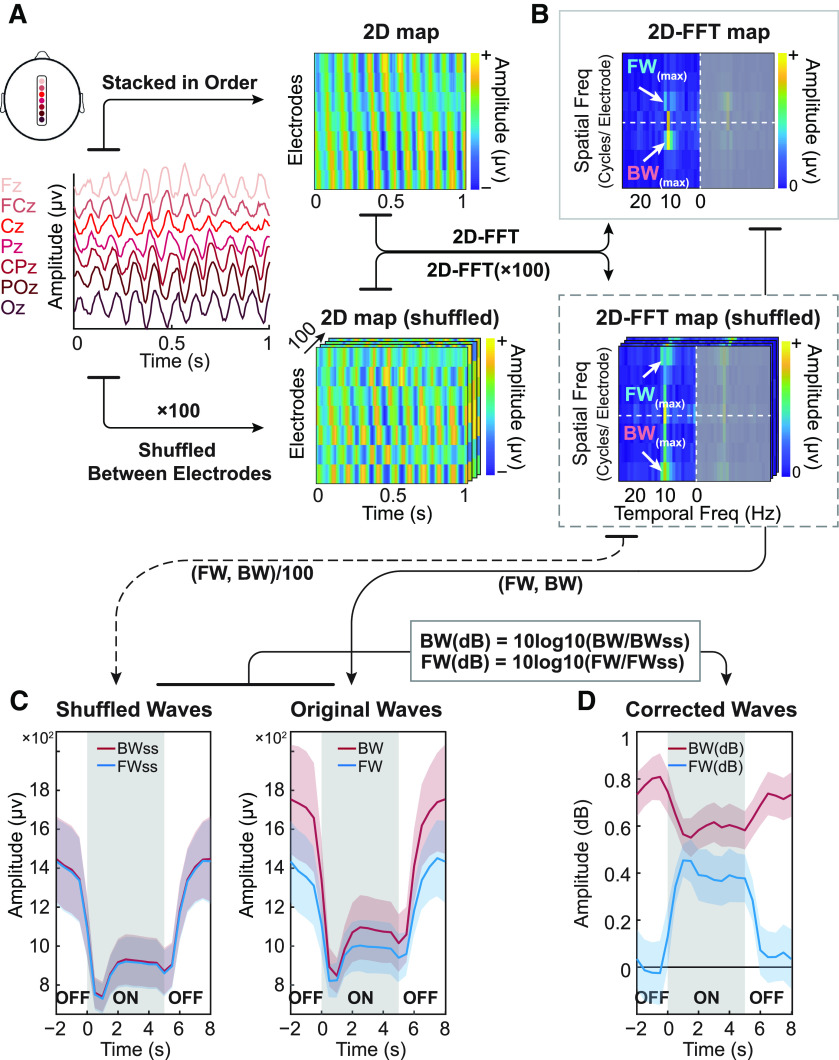
α Band traveling waves in raw EEG signals. ***A***, left, Seven midline electrodes of the 10–20 system are ordered from posterior to anterior (Oz to Fz) and backward traveling waves (BW) can be observed in the 1-s-long time window. Right, A 2D map of the same data with electrodes stacked in order and with amplitude color coded (top). To statistically quantify the waves’ direction, we employed a non-parametric test by shuffling the electrodes’ order for each time window 100 times. The resultant surrogate 2D maps eliminate the spatial structure of the original signals, including their original propagating direction (bottom). ***B***, Temporal frequencies (*x*-axis) and spatial frequencies (*y*-axis) for both real and surrogate data are obtained by computing a 2D-FFT. The temporal frequencies were computed up to 80 Hz, but only displayed until 25 Hz for illustration purposes. Since the 2D-FFT gives symmetrical results around the origin, we only focused on the left part of the plot. The maximum value in the upper quadrant represents the strength of forward traveling waves, while the maximum value in the lower quadrant quantifies the strength of feedback traveling waves. ***C***, left, For surrogate data, we averaged the 100 surrogate values separately for BW and FW signals and for each time bin. Colored shaded area stands for SEM across subjects; the stimulus-on period was shifted to the center part (gray shaded area) for better visualization of these dynamics around stimulus onset and offset. Right, Similar time courses were obtained for the real data. ***D***, The surrogate line plots were used as a baseline, mostly reflecting the background (α) oscillatory power. After correcting for these baseline fluctuations (and expressing the result in dB, as per the equations), we obtained a measure of the dynamics of FW and BW waves. Colored shaded area stands for SEM across subjects.

In order to quantify the presence of traveling waves in EEG signals and assess the propagation direction, we adopted a wave quantification method from our precious studies (Alamia and VanRullen, 2019; Lozano-Soldevilla and VanRullen, 2019), which is described in Figure 2. For each subject, every trial (10 s long with 0.5-s baseline) was divided into 20 time bins by a sliding window of 1 s (with 500-ms overlap). For each time bin, we stacked EEG signals from seven midline electrodes (from posterior to frontal: Oz, POz, Pz, CPz, Cz, FCz, Fz) to form a 2D (electrode-time) map. To computationally quantify the waves’ amount, we used a 2D-FFT (2-D fast Fourier transform) transform for each 2D map. This transform results in temporal frequencies along the horizontal axis as well as spatial frequencies along the vertical axis. The horizontal midline indicates stationary oscillations with no spatial propagation, while the upper and bottom quadrants reflect forward-propagating (FW) and backward-propagating (BW) waves, respectively. We extracted the max value within the α band temporal frequencies (8–13 Hz) from the upper quadrant of the 2D-FFT as the FW value for this time window, and the max value (also within the α band) from the lower quadrant as the BW value. After repeating this procedure over all 20 time bins, we finally obtained two curves representing the dynamic changes of FW and BW waves along time.

To assess statistical significance of traveling waves, we used a non-parametric test. Specifically, we shuffled the electrodes’ order 100 times for each time bin, thereby eliminating any spatial organization of the oscillatory signals (including traveling waves). For this surrogate data set, we repeated the same 2D-FFT procedure as described above. Since the shuffling procedure only eliminated the spatial structure but left intact the oscillatory power of EEG signals, the resulting FW and BW curves (Fig. 2*C*), based on the maximum power in each quadrant, could still fluctuate across time: oscillatory power was relatively suppressed during stimulus-on periods, then increased in the absence of visual input. These power fluctuations in the surrogate data, however, were similar in the FW and BW directions (as expected because of the shuffling procedure). In order to focus on the differences between real and surrogate data, we corrected the real wave patterns by dividing their values by the corresponding surrogate patterns, and expressing the result in dB units (Fig. 2*D*).

### Analysis

We first conducted one-sample *t* tests against zero for both BW and FW waves separately to confirm their presence at each time point [corrected for multiple comparisons via false discovery rate (FDR), α = 0.05]. Second, we examined differences between the waves across the different experimental conditions. For this, we conducted a within-subject three-factor repeated measure ANOVA: CONDITIONS (static vs dynamic visual stimulation) × WAVES (FW vs BW) × TIME BINS (20). To clearly examine the influence of tasks (visual processing vs rest state), we also grouped all time points within the stimulus-on and stimulus-off periods and conducted another ANOVA with factors CONDITIONS (static vs dynamic) × WAVES (FW vs BW) × TASKS (stimulus-on vs stimulus-off).

## Results

### Stimulus-evoked FW waves and spontaneous ongoing BW waves

**Figure 3.**
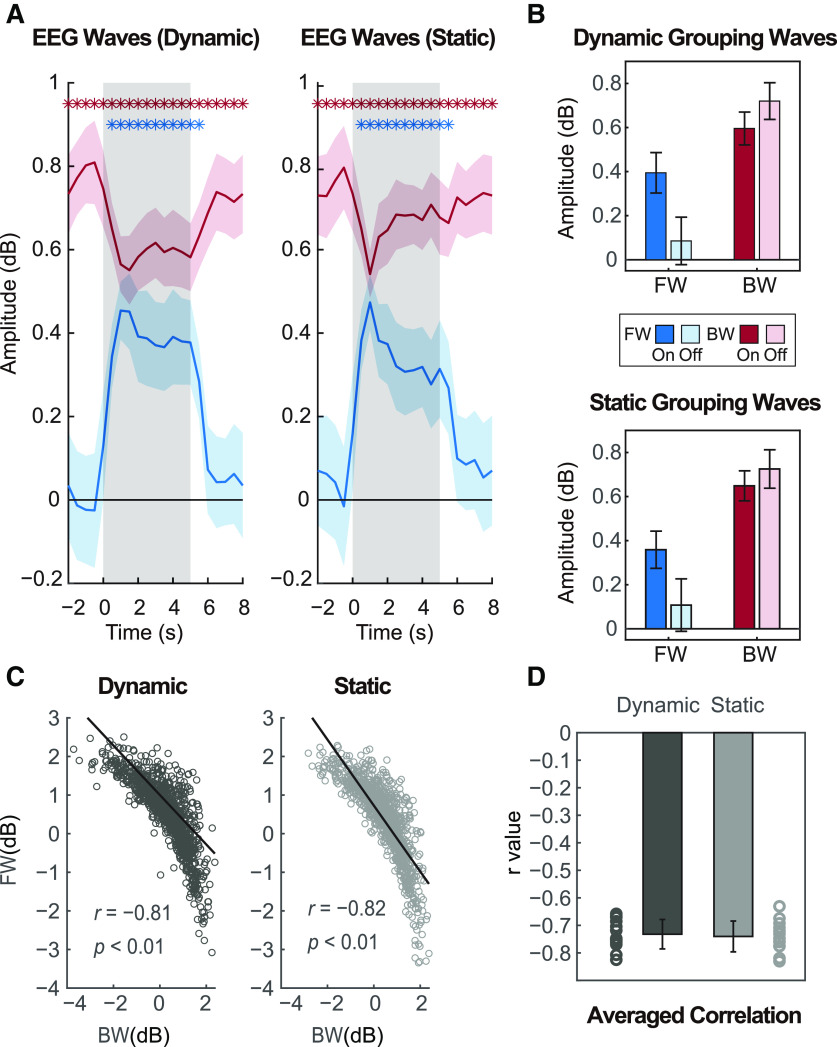
Amplitude of FW and BW waves and their correlation. ***A***, The two plots show how waves evolve over time under dynamic (white-noise visual sequences) and static (full contrast visual sequences) task conditions. Blue and red asterisks represent separately the significant time points (corrected for multiple comparisons by FDR) for FW waves (blue) and BW waves (red) when compared with zero. ***B***, To better compare wave patterns, waves were grouped over time points within each period type (stimulus ON/OFF). ***C***, For stimulus-on periods, we computed the correlation between BW and FW values under dynamic stimulation (left) and static stimulation (right). The two scatter plots show results from a representative subject. Each dot represents a pair of FW and BW values for each single time bin (1 s) and black lines are regression lines. ***D***, Bar graphs show averaged correlation (r values) across all subjects for dynamic and static conditions. The corresponding circles are individual results.

Figure 3*A* illustrates the evolution of FW (blue) and BW (red) waves as a function of time under dynamic and static visual stimulation conditions, averaged across all subjects. The waves’ traces in both plots show similar patterns overall: BW waves are relatively high during both stimulus-on and stimulus-off periods (with a decrease during stimulation), while FW waves seem to only emerge after the onset of visual stimulation, and disappear after the offset of visual inputs. In other words, the occurrence of FW waves is highly dependent on external stimulation while BW waves exist both in the presence and absence of stimulation. Therefore, we propose that FW waves are associated with visual processing (e.g., as a stimulus-evoked wave), while BW waves reflect ongoing spontaneous or endogenous activity. To support this, we conducted one-sample *t* tests against zero (*p *<* *0.05, corrected for multiple comparisons by FDR) for the two waves separately at each time point. BW waves were significant during the entire time course (significant values are marked with asterisks in Fig. 3*A*). However, FW waves were only significant from 0.5 to 5.5 s in both dynamic and static conditions.

**Figure 4.**
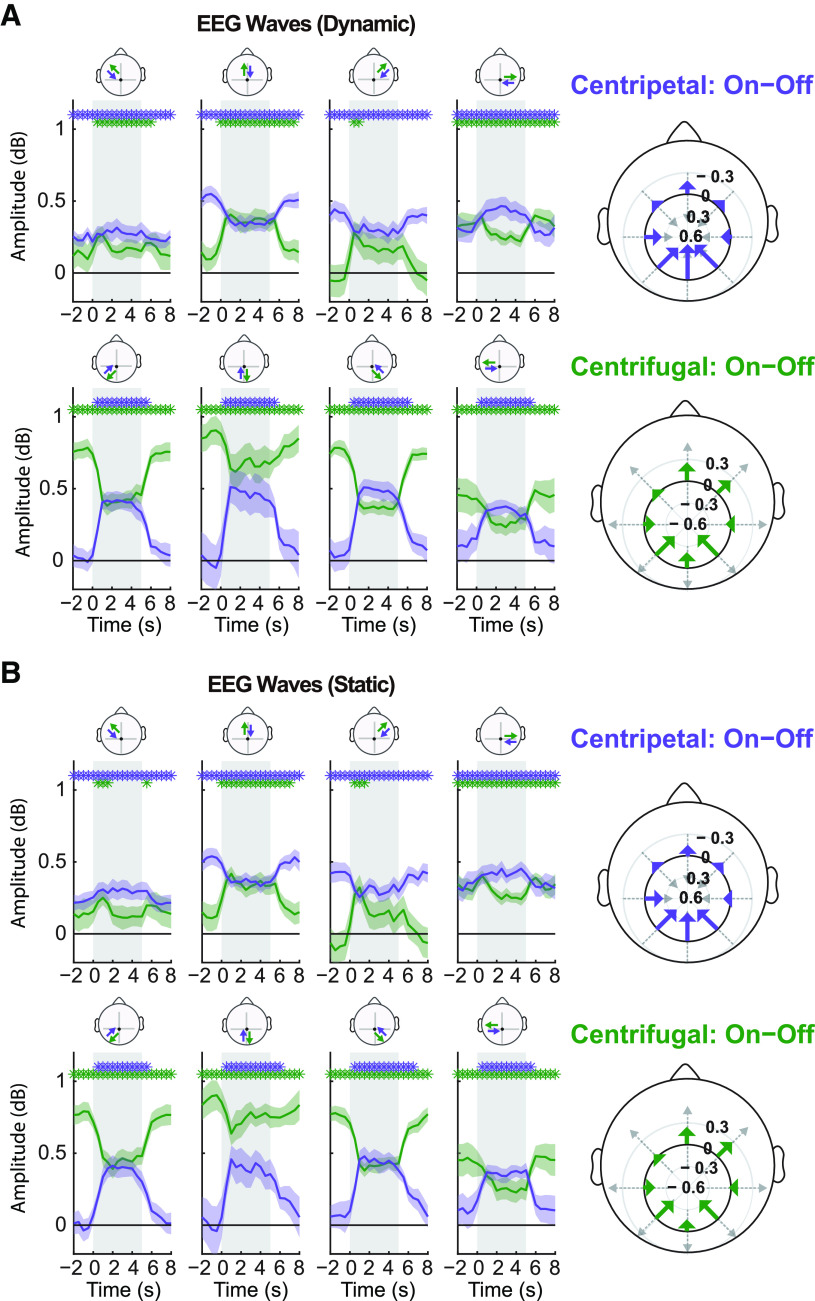
Traveling waves across the scalp. ***A***, left, Changes of wave amplitude over time under dynamic stimulation conditions. Around the central electrode CPz, we selected eight lines of electrodes to cover the whole scalp. As before, we computed two waves in opposite directions for each path (see inset topography above each subplot); here they are sorted as centripetal (toward CPz, in purple) and centrifugal (away from CPz, in green) waves. Each path counted only five electrodes, which were interpolated into seven sampling points, so as to remain comparable with our main analysis (see Fig. 3*A*). Right, Polar representation of the difference of wave amplitude between stimulus-on (gray shaded regions in left subplots) and stimulus-off period (white regions). Each wave is represented as an arrow, centered and aligned on the corresponding path, with a length proportional to its amplitude difference (with negative differences pointing in the opposite direction). The top image represents the vector field for centripetal waves, the bottom one for centrifugal waves. In both cases, the net effect of visual stimulation is to increase the forward propagation of the waves (and/or decrease their backward propagation, with little effect along the lateral axes). ***B***, Traveling waves under static stimulation conditions (notations and conclusions as in ***A***).

While the directionality of the waves along the occipito-frontal midline appears clear, one might wonder whether and how waves propagate over the rest of the scalp. Figure 4 illustrates the directional propagation along eight distinct paths on the scalp. Specifically, eight lines of five electrodes each were selected around the central electrode CPz. Since we could derive two opposite waves along each line, and not all of them included a clear FW versus BW direction (i.e., lateral lines), we sorted the waves in two groups: centripetal waves, toward CPz (purple), and centrifugal waves, away from CPz (green). Both the dynamic and static conditions showed very similar patterns: all the waves running in the occipito-frontal direction, including vertical but also diagonal lines, showed relatively large and task-dependent fluctuations, going from near-zero amplitude during rest to strong positive values during stimulation. The opposite (fronto-occipital) direction in each line tended to show significant waves throughout each trial, but stronger during rest and decreasing during visual stimulation. Finally, lateral directions of propagation displayed the smallest amount of fluctuations, regardless of the (centripetal or centrifugal) direction. This overall pattern was further confirmed by the polar plots on the right of the figure, obtained by subtracting averaged waves during stimulus-off periods from the corresponding waves during stimulus-on periods. In all cases, the changes in wave amplitude caused by the visual stimulation onset mainly lie along the occipital-frontal direction. Thus, our initial result with midline electrodes appears to be representative of the behavior of traveling waves across the entire scalp.

### Both FW and BW waves are task dependent

After establishing the presence of FW waves during visual stimulation, and BW waves during both visual stimulation and resting state, we further examined the properties of both waves under various task conditions, using a three-way repeated measures ANOVA with factors CONDITIONS (dynamic/static), WAVES (FW/BW) and TIME BINS (20 values). This revealed main effects for TIME BINS (*F*_(19,228)_ = 19.083, *p *<* *0.001, $\eta_{\text{p}}^{2}$ = 0.614) and WAVES (*F*_(1,12)_ = 7.048, *p *=* *0.021, $\eta_{\text{p}}^{2}$ = 0.37), a significant two-way interaction for WAVES × TIME BINS (*F*_(19,228)_ = 9.002, *p *<* *0.001, $\eta_{\text{p}}^{2}$ = 0.429), as well as a significant three-way interaction (*F*_(19,228)_ = 3.103, *p *<* *0.001, $\eta_{\text{p}}^{2}$ = 0.205).

The CONDITIONS × TIME BINS interaction reached significance for FW waves (*F*_(19,228)_ = 2.698, *p *<* *0.001, $\eta_{\text{p}}^{2}$ = 0.184) at time points 2, 3.5, and 5 s, and for BW waves (*F*_(19,228)_ = 2.928, *p *<* *0.001, $\eta_{\text{p}}^{2}$ = 0.196) at time points 2–4 and 5 s. That is, the time course of FW waves showed less power for static stimulation at certain time points. BW waves were also influenced by the stimulation type but with increasing power for static stimulation toward the later part of each stimulation period (Fig. 3*A*). We speculate that both waves may be influenced by stimulus complexity since static stimuli are much simpler and more predictable compared with dynamic white-noise luminance sequences.

FW waves were only present during visual processing, while BW waves existed during both task conditions (visual processing vs rest). To further examine whether BW waves showed significant differences associated with the tasks, another three-way repeated measures ANOVA was conducted with factors CONDITIONS (dynamic/static), WAVES (FW/BW) and TASKS (stimulus-on/off). That is, the TIME BINS factor (20 values) was replaced with the TASKS factor (two values). The wave amplitudes were averaged over time bins (separately for the stimulus-on and stimulus-off conditions). This time, we did not obtain a significant three-way interaction (Fig. 3*B*). Instead, the ANOVA revealed a significant two-way interaction for WAVES × TASKS (*F*_(1,12)_ = 18.056, *p *=* *0.001, $\eta_{\text{p}}^{2}$ = 0.6). As expected, the main effect of CONDITIONS was significant (*F*_(1,12)_ = 25.95, *p *<* *0.001, $\eta_{\text{p}}^{2}$ = 0.684) for FW waves, similar to the result of one-sample *t* tests above (Fig. 3*A*). For BW waves, the main effect of CONDITIONS was also significant (*F*_(1,12)_ = 7.196, *p *=* *0.02, $\eta_{\text{p}}^{2}$ = 0.375) with higher BW power in the absence of visual inputs. The modulation of BW waves by visual stimulation is in line with other studies showing that spontaneous traveling waves could be suppressed by external inputs (Patten et al., 2012; Sato et al., 2012).

In summary, FW waves appear to be caused by external visual stimulation, while BW waves can originate spontaneously but could be reduced (yet not eliminated) by the presence of visual stimulation. During visual stimulation, both waves are present and modulated by the type of visual inputs, with lower FW but higher BW waves’ power for simpler (static) sensory stimulation.

### FW and BW waves are negatively related during visual stimulation

During visual stimulation (stimulus-on periods), both FW and BW waves appear to be simultaneously present (Fig. 3*A*). However, the average traveling wave’s behavior does not necessarily reflect the instantaneous state of the brain and its dynamics: FW and BW waves may by truly equally present at each moment in time, or they may tend to happen in alternation, at distinct moments in time. To further examine the relationship between them, we assessed the moment-to-moment correlation between FW and BW waves for each stimulus condition. Figure 3*C* shows scatter plots from a representative subject. For each condition, each 1-s time bin window produces one pair of FW and BW wave values, i.e., a single dot in the plot. To discard the common influence of oscillatory amplitude fluctuations on both FW and BW traveling waves, we correct each wave’s value by its corresponding averaged surrogate value (obtained by shuffling the electrodes’ order 100 times, as explained in Fig. 2*A*). The correlation between the resulting FW and BW values in dB units showed a clear and significant (*p *<* *0.01) negative trend, for both the dynamic and static stimulus conditions. This means that when FW waves were stronger, BW waves tended to be weaker, and vice-versa. Figure 3*D* gives the average correlation across all subjects: significant negative correlation between FW and BW waves can be observed for both the dynamic (mean = −0.732 ± 0.053, *t*_(12)_ = −49.371, *p *<* *0.001, 95% confidence interval (CI): −0.765 to −0.7) and static conditions (mean = −0.74 ± 0.056, *t*_(12)_ = −47.644, *p *<* *0.001, 95% CI: −0.774 to −0.706).

## Discussion

Based on EEG data from human participants, we demonstrated that the direction of α traveling waves (8–13 Hz) is task dependent, confirming suggestions from prior studies (Zhang et al., 2018; Alamia and VanRullen, 2019; Halgren et al., 2019; Lozano-Soldevilla and VanRullen, 2019), and verifying the predictions of our own modeling study on the generation and propagation of α oscillations (Alamia and VanRullen, 2019). Specifically, we characterized FW waves traveling from occipital to parietal regions elicited by visual stimulation, and BW waves in the reversed direction dominating during rest state. Furthermore, the presence of external visual stimulation reduced BW waves (Fig. 3*A*), which is in line with other studies on spontaneous traveling waves (Patten et al., 2012; Sato et al., 2012). Lastly, during visual stimulation, FW waves and BW waves were present and modulated by stimulation type (static or dynamic), but they were negatively correlated over time.

### Contributions of the current study

It should be emphasized that the current experimental design directly contrasted the conditions of visual processing and resting state within each trial. Previously, a number of studies had examined traveling waves under various single-task conditions, including visual stimulation (Nauhaus et al., 2012; Muller et al., 2014; Alamia and VanRullen, 2019; Lozano-Soldevilla and VanRullen, 2019), sleep (Muller et al., 2016), or quiet wakefulness (Alamia and VanRullen, 2019; Halgren et al., 2019). While these experiments confirmed the existence of traveling waves, they did not make it possible to track how the waves change from one condition to another. Because of the within-subject design in the present study, we found that the waves’ direction is highly sensitive to the task conditions.

Compared with previous studies using dynamic white noise sequences as visual stimulation (Alamia and VanRullen, 2019; Lozano-Soldevilla and VanRullen, 2019), we also included a simpler type of visual stimulation: static luminance sequences. The results showed that although these two stimulus types evoked similar FW and BW waves, toward the later stages of visual stimulation, the BW wave power increased at time points 2–4 and 5 s and FW wave power decreased at 2, 3.5, and 5 s. This may be because of the relative simplicity of static inputs compared with the dynamic ones. The same factor, and the longer trial durations, may also explain why we measured less FW power overall compared with our prior studies (Alamia and VanRullen, 2019; Lozano-Soldevilla and VanRullen, 2019). Simpler stimuli and longer trials may result in subjects being less engaged in the task, and consequently in weaker FW waves overall. Future studies should explore whether the overall amount of FW power can be increased by parametric manipulations of the task or of the experimental stimuli or screen background.

Unlike prior studies measuring a single traveling wave direction from phase gradients over certain brain regions (Zhang et al., 2018; Halgren et al., 2019), we here derived two opposite components of the waves’ direction from the pattern of brain activity within each time window, and quantified their strength. Previous studies have shown that traveling waves can propagate in different directions (Alexander et al., 2009; Patten et al., 2012), and that the co-existence of two opposite waves may cause a loss of wave information (Alexander et al., 2013). For example, under cortical states like sleep or resting state, traveling waves have often been reported to propagate in a frontal-to-occipital direction (Massimini et al., 2004; Alamia and Vanrullen, 2019). However, traveling waves are less frequently observed under more complex cognitive states (Alexander et al., 2013), this may be caused by the interference of waves propagating in opposite directions, while their direction is characterized as a single value. Instead, our analysis method used in the current study independently quantifies waves propagating in the two directions. Also, the separation of FW and BW waves’ components contributed to reveal their distinct functional roles. We revealed a closer link between FW waves and visual processing as an evoked wave, since FW waves emerged at the onset of visual input and disappeared right after the offset (Fig. 3*A*); meanwhile, BW waves were more related to the resting state, acting as a spontaneous wave.

### An explanation under the predictive coding framework

The generation and directionality of traveling waves can tentatively be interpreted within the predictive coding framework (Rao and Ballard, 1999). In our previous work (Alamia and VanRullen, 2019), researchers built a seven-level hierarchical model of visual cortex with bidirectional connectivity implementing predictive coding. Within the hierarchy, higher levels predicted the activity of lower ones through inhibitory feedback, and lower levels sent the prediction error via feedforward excitation to the higher layers to correct their prediction. With biologically plausible parameters (neural time constants, communication delays), this model produced α rhythms traveling through the hierarchy. The waves could travel in the FW direction when the model was presented with visual inputs, and in the BW direction in the absence of inputs (while the model was processing “top-down priors” instead of bottom-up sensory signals).

In this context, it is reasonable to infer that FW waves carry “residual error” signals (the difference between the actual visual inputs and the prediction from higher-level regions), while BW waves carry the prediction signals. Remarkably, the current results that FW waves emerged only during visual stimulation and BW waves were dominant in the resting state agree with this framework. On the other hand, the negative correlation across time between FW waves and BW waves during visual stimulation may reflect the dynamics of predictive coding mechanism. That is, stronger prediction signals within BW waves are associated with weaker prediction errors carried by FW waves and vice versa. Moreover, in the static condition, BW waves increased but FW waves decreased significantly at the later stages of visual stimulation, indicating that prediction information becomes stronger while error signals weaken over time. This was not the case in the dynamic condition, which has much more complex (and unpredictable) stimulus temporal structure, leading to less precise prediction signals and larger error signals.

### Spontaneous BW waves may reveal top-down control

Spontaneous ongoing waves have been reported in the cortex under anesthesia or quiet wakefulness (Petersen et al., 2003; Sakata and Harris, 2009; Alamia and VanRullen, 2019). The current study points to BW waves as spontaneous waves, given their existence under resting state. Besides, the significant reduction of BW waves because of the presence of visual inputs also agrees with other studies on spontaneous traveling waves (Patten et al., 2012; Sato et al., 2012). This reduction could be explained by the desynchronization caused by visual processing, since spontaneous activity measured during quiet wakefulness may reflect synchronized cortical states (Harris and Thiele, 2011). On the other hand, given the spatial extent of traveling waves across distributed cortical regions, their functional role may entail long-range information integration (Sato et al., 2012; Halgren et al., 2019). In particular, it is speculated that BW waves may participate in the organization of top-down or feedback information flow (Van Kerkoerle et al., 2014; Halgren et al., 2019). This is in line with the dominance of α band activity in the waves, a frequency which is typically associated with top-down control (Klimesch et al., 2007b; Jensen et al., 2012).

### Stimulus-evoked FW waves are associated with bottom-up sensory processing

In the current study, we measured α FW waves which were directly linked with visual processing (and absent during rest). This direct link is also supported by our prior studies of perceptual echoes: since these echoes are measured by cross-correlation with the visual input sequence, they can be viewed as a direct reflection of visual processing (VanRullen and Macdonald, 2012; Alamia and VanRullen, 2019; Lozano-Soldevilla and VanRullen, 2019). Recent work from our group found that these perceptual echoes propagate from occipital to parietal regions in a forward direction (Alamia and VanRullen, 2019; Lozano-Soldevilla and VanRullen, 2019). Although further research is needed to test whether FW waves also contribute to sensory processing in other modalities (like audition or touch), FW waves may serve to integrate the information flow along the bottom-up path. This is consistent with the “scanning hypothesis” proposed by Pitts and McCulloch (1947), suggesting that the α rhythm repeatedly scans the visual cortex. The bidirectionality of α traveling waves found in the current study may help to clarify an apparent contradiction between the conventionally postulated inhibitory role of α oscillations (Jensen and Mazaheri, 2010; Bonnefond and Jensen, 2012), and their reported implication in sensory processing (Varela et al., 1981; VanRullen, 2016). Inhibition may be carried by the BW component of α oscillations as mentioned above, whereas, the FW component may reflect the positive relation between α and sensory processing.

In summary, the current study corroborated the predictions from our prior EEG and modeling study (Alamia and VanRullen, 2019). It showed that FW and BW waves are inversely related to sensory processing, and may characterize opposite directions of information flow in the brain hierarchical system. Importantly, the transitions between FW and BW waves were observed within single trials and for the same human subjects. First, FW waves travel from occipital to frontal regions during visual processing, while BW waves are spontaneously generated and travel in the opposite direction, likely reflecting a feedback process. Second, during visual stimulation, both FW and BW waves exist on average, but are negatively correlated across time, suggesting that they reflect distinct functions that may draw on common brain resources.

## Synthesis

Reviewing Editor: Bradley Postle, University of Wisconsin Decisions are customarily a result of the Reviewing Editor and the peer reviewers coming together and discussing their recommendations until a consensus is reached. When revisions are invited, a fact-based synthesis statement explaining their decision and outlining what is needed to prepare a revision will be listed below. The following reviewer(s) agreed to reveal their identity: Joshua Jacobs, Ali Mazaheri.

In this paper the authors examine the phenomenon of cortical traveling waves in the human brain, examining scalp EEG recordings from subjects performing a perceptual task. Extending earlier work, they examined how the timing of waves moved in different directions in relation to the timing of stimulus presentation in the task. The main findings were that alpha oscillations were traveling waves that moved in different directions throughout the task, with forward waves being relatively more prominent when stimuli were presented. Overall, backwardly propagating waves were more common. These results are important because they suggest a new role for the functional properties of traveling waves and, as the authors mention at the end of the paper, suggest a potential new interpretation for the fundamental role of alpha oscillations by showing that they can perform multiple roles based on their function.

Overall the important findings from this concise paper are nicely described and the data analyses support the authors’ interpretations. I have some minor suggestions for improving the results.

* The paper should give a more complete description and interpretation of the fact that although there were changes in the relative prevalence of traveling waves in different directions over time, it still is the case that overall backward waves are much more prominent at all timepoints. It would be useful to know the authors’ interpretation of this fact, which seems in some ways at odds with their main conclusions. Would a different task, or other methodological changes, have shown that sometimes forward waves actually became more prominent than backwards ones?

* The authors used a simple analytic technique of only analyzing electrodes in the midline (1D). This means that they could not assess any laterality in the direction of traveling waves nor truly assess their velocity or wave length in 2D/3D. They might discuss the implications of this choice because their results are not, as a result, able to distinguish waves that move directly in a posterior-anterior direction from those that are mostly lateral with just a small A-P component.

* In between stimuli the the task had a black screen. Earlier work shows that viewing very dark scenes can give rise to feed forward input and arguably a gray screen could have been useful to more strongly elicit top-down responses by inactivating visual cortex. The authors might discuss this because using different stimuli could possibly even strengthen their results.

* Figures 2C/D could be improved by using similar formatting/scales across both plots.

* The scatter plots in Figure 4 are not described fully. Does each point represent one trial? At what timepoint is each value computed at?

* The paper’s title is a bit strange grammatically. I suggest revising it to improve how it sounds in english.

R2’s summary for editor:

A number of previous studies have found evidence for the presence of traveling waves. The current study goes a step further and directly contrasts the direction of traveling waves in a participant between a condition of rest versus visual stimulation. They rather intriguingly demonstrate the waves go in different directions.

The Contributions of the current study

314 It should be emphasized that the current experimental design directly

315 contrasted the conditions of visual processing and resting state within each

316 trial. Previously, a number of studies had examined traveling waves under

317 various single-task conditions, including visual stimulation (Alamia &

318 VanRullen, 2019; Lozano-Soldevilla & VanRullen, 2019; Muller et al., 2014;

319 Nauhaus et al., 2012), sleep (Muller et al., 2016) or quiet wakefulness (Alamia

320 & VanRullen, 2019; Halgren et al., 2019). While these experiments confirmed

321 the existence of traveling waves, they did not make it possible to track how the

322 waves change from one condition to another. Due to the within-subject design

323 in the present study, we found that the waves’ direction is highly sensitive to

324 the task conditions.

R2’s Comments to authors: This was a really interesting study. While the sample size was on the lower end, I find it rather amazing that they were able to observe this effect. This suggests the results are rather robust.
